# On the use of inexact, pruned hardware in atmospheric modelling

**DOI:** 10.1098/rsta.2013.0276

**Published:** 2014-06-28

**Authors:** Peter D. Düben, Jaume Joven, Avinash Lingamneni, Hugh McNamara, Giovanni De Micheli, Krishna V. Palem, T. N. Palmer

**Affiliations:** 1 Atmospheric, Oceanic and Planetary Physics, University of Oxford, Clarendon Laboratory, Parks Road, Oxford OX1 3PU, UK; 2 Integrated Systems Laboratory (LSI), Ecole Polytechnique Fédérale de Lausanne (EPFL), 1015 Lausanne, Switzerland; 3 Department of Electrical and Computer Engineering (ECE), Rice University, 6100 Main Street, Houston, TX 77005, USA; 4 Mathematical Institute, University of Oxford, Andrew Wiles Building, Radcliffe Observatory Quarter, Woodstock Road, Oxford OX2 6GG, UK

**Keywords:** energy efficient computing, Lorenz ’96, inexact hardware design, scale separation

## Abstract

Inexact hardware design, which advocates trading the accuracy of computations in exchange for significant savings in area, power and/or performance of computing hardware, has received increasing prominence in several error-tolerant application domains, particularly those involving perceptual or statistical end-users. In this paper, we evaluate inexact hardware for its applicability in weather and climate modelling. We expand previous studies on inexact techniques, in particular *probabilistic pruning*, to floating point arithmetic units and derive several simulated set-ups of pruned hardware with reasonable levels of error for applications in atmospheric modelling. The set-up is tested on the Lorenz ‘96 model, a toy model for atmospheric dynamics, using software emulation for the proposed hardware. The results show that large parts of the computation tolerate the use of pruned hardware blocks without major changes in the quality of short- and long-time diagnostics, such as forecast errors and probability density functions. This could open the door to significant savings in computational cost and to higher resolution simulations with weather and climate models.

## 1. Introduction

Despite steady increases in the performance of state-of-the-art supercomputers, the available computing resources still cannot satisfy the demand for computational power. For some time now, the main increase in FLOPS^1^ of today's computing centres is not so much caused by an increase of performance of a single processor, but rather by an increase of the number of processors that run in parallel. The work with 10^6^ or 10^7^ processor cores in one supercomputer brings several challenges for both the development and use of high-performance computing facilities. Two main challenges are the high energy demand and error-resilience. Plans to build a computer capable of ‘exascale’ performance (approx. 10^18^FLOPS) warn both that ‘traditional resiliency solutions will not be sufficient’ and that typical power supply limits (of about 20 MW) will not be met [1].

The increasing costs of power are beginning to force hardware developers to rethink some of the principles of computing. One candidate is to trade high precision or even the reproducibility of computations for reduced energy demand and/or higher performance. Over the past decade, a variety of approaches have been proposed to take advantage of the error-resiliency in several current and emerging classes of applications, in particular media/signal processing and recognition, mining and synthesis. These approaches advocate trading the accuracy of the underlying hardware fabric in return for significant savings in the hardware resources used such as energy, delay, area and/or yield and, therefore, lead to a reduced cost for computing. Dubbed inexact [2] or approximate computing, this work has now led to a subfield of active research spanning methodologies that exploit the fact that, quite often, applications do not need to have *precise* outputs. Taking advantage of various inexactness-inducing ‘knobs’ to vary the hardware quality at different levels of hardware design abstraction, our own work has shown that these inexact methodologies could result in significant resource savings in exchange for entirely tolerable accuracy trade-offs. The feasibility of these resource–accuracy trade-offs has been successfully demonstrated in several key resource-intensive arithmetics and digital signal processing primitives [3,4].^2^

Several techniques at different levels of hardware design abstraction have been proposed to realize inexact hardware. Physical/circuit-layer techniques such as voltage overscaling and its variants have been the popular choice in the beginning to induce inexactness [5–7]. Later, owing to the ease of hardware realization, inexact techniques moved towards higher levels of abstraction such as the logic/architecture layers [4,8]. In this paper, we focus on one of these inexact design techniques, *probabilistic pruning* [8], that, apart from its implementation ease, has been shown to achieve significant gains in all of energy, delay and area in exchange for tolerable amounts of accuracy loss demonstrated in the context of integer arithmetic units. However, in order to extend and explore the inexact design techniques to a broader milieu of computing encompassing general-purpose processors and high-performance workloads, this existing work on pruning would require the extension to floating point units, an aspect that has not received much attention so far.

The weather and climate modelling community is a heavy user of high-performance computing, and weather and climate models run on supercomputers that are among the fastest in the world. Even so, the model resolution is far from being adequate [9] and limited by the available computing power. An increase in computational power would allow higher resolution simulations and produce higher quality weather and climate predictions.

A recent study [10] investigated the use of inexact hardware in weather and climate modelling. Faulty or low precision hardware was emulated within simulations of a simple atmosphere model based on spectral discretization methods to investigate the sensitivity of various components of the model to hardware-induced errors. The study revealed that large parts of a model integration can be computed on inexact hardware without serious penalties, provided the sensitivities are respected, for example by using low precision for small-scale dynamics and high precision for large-scale dynamics [10].

It is the aim of this paper to initiate a successful cooperation between the two scientific communities of inexact hardware development and weather and climate modelling. We expand previous studies on pruning techniques to floating point arithmetic units (FPUs). These pruning techniques are used to design FPUs with a wide range of accuracy degradations. We test the applicability of this hardware in atmospheric modelling by emulating the use of the pruned hardware in simulations of the Lorenz ‘96 model, a toy model for atmospheric dynamics, and test the sensitivity of different parts of the model to reduced precision FPUs. The emulation is configured by measuring error patterns of the FPU designs for inputs typical of the Lorenz ‘96 simulations. The results of these simulations are used to further refine the hardware designs, increasing or reducing the errors as allowed or required. This iterative design loop was repeated several times. We wish to emphasize that throughout this paper, we might use the word hardware for convenience to refer to simulations of synthesized versions of FPUs as opposed to fabricated integrated circuits.

We present results for simulations with 10 FP adder–subtractor and 10 FP multiplier blocks and list the expected savings for area and power consumption and the increase in performance compared with a precise double precision FPU for each set-up. After preliminary tests for which we compute only one subroutine of the model with the emulated pruned hardware, we decide on four combinations of FP adder–subtractor and multiplier blocks that are used to identify the sensitivities to hardware faults of the different parts of the model. Finally, we try to simulate as many parts of the model as possible without serious penalties.

Section 2 gives details on pruning and the development of pruned FPUs with reasonable error rates. Section 3 provides a detailed description of the Lorenz ’96 model and the emulator which mimics inexact hardware. Section 4 presents the derived hardware set-ups that are simulated, the results of numerical simulations and a cost estimation for the different simulations.

## 2. Inexact hardware design

Here, we describe the methodology for the design of the inexact FPU. As this paper is meant to be a first approach only, we limit ourselves to a simple inexact design technique to demonstrate the utility of such inexact hardware for the targeted atmospheric modelling application and defer the exploration of more complicated approaches involving the usage of multiple inexact design techniques from different layers of design abstraction simultaneously [11] to future papers. As mentioned in §1, we chose *probabilistic pruning* [8] as our inexact technique given its ease of implementation and the ability to provide *zero hardware overhead* realizations. The pruning algorithm is revisited in §2*a* and its usage in the context of FPUs is described in §2*b*.^3^

### (a) Methodology for inexact design

The main idea behind pruning is to reduce the size of a hardware architecture by removing parts that are hardly used or do not have a significant influence on the calculations. We consider a circuit that implements a floating-point binary operation (such as addition or multiplication). This circuit consists of logic gates connected by wires, with each gate accepting (binary) inputs and producing (binary) outputs. Our probabilistic pruning algorithm [8] operates by building a directed acyclic graph of the circuit with nodes denoting a gate or a collection of gates and edges denoting the interconnections. It then annotates each of the nodes in the circuit with analytically or empirically derived *significance* and *activity* values. The significance value quantifies the impact of a node on the accuracy of the circuit. A node which is only connected to a least significant bit of the output has a lower value than a node connected to a bit of higher significance. To this end, the significance value depends on the circuit topology. The activity value denotes the number of times the output(s) of a node switch between the two possible binary values (a 0 or a 1). The activity value, to some extent, depends on the application being run, for example if input values of a specific node are biased towards either 0 or 1 for the given application. Using these annotations on each of the node as a basis, the pruning algorithm proceeds through two main steps:

1. *Ranking phase*. In this step, each of the node in the circuit is ranked (in the order to be pruned) using a function of the significance and activity values. We choose the significance-activity product (SAP) as a basis for ranking the nodes. In our usage, the nodes with higher SAP values receive a lower rank (hence, lower likelihood of being pruned away).
2. *Pruning phase*. Equipped with the desired error metrics, the pruning step works on deleting the nodes in the circuit that have the highest rank. In this step, we use the knowledge we have on the statistics of the activity of each node. If one of the input values is very likely to be observed at the output of the pruned node, then we use a *greedy*^4^ substitution.^5^

Once we have reached the targeted error bound,^6^ we re-synthesize the circuit to eliminate any redundant logic and evaluate the resource savings achieved as a result of this trade-off.

### (b) Implementing an inexact floating point unit

The most important aspect of pruning involves the annotations of the node with the significance and activity values. Hence, in order to better configure the inexact hardware for the targeted application, we require input traces that capture the statistics of the application and guide the pruning algorithm. In this paper, as we are targeting the arithmetic units with regular structures that stay fairly generic, we use the output significance level to determine the significance of the nodes (similar to earlier works on inexact adders [12]), i.e. nodes connected to the most significant bits of the outputs receive higher significance values.

In this paper, we extend previous work [8] to include the FPU. We applied pruning in the *significand*. To enable the pruning techniques, and in order to simulate inexact behaviour of different architectures, we designed a bit width parametrizable (up to 64-bits—double precision) FPU which is compliant with the IEEE-754 standard [13] using VHDL/Verilog hardware description languages. The FPU co-processor architecture is composed by a dual pipelined execution unit, (i) for addition/subtraction operations, and (ii) for multiplication operations. Thus, each double precision addition/subtraction operation can be executed in parallel with each multiplication. The architecture is similar to the approaches presented in references [14,15]; however, in this work, the design of the FPU has been optimized to combine high-throughput with low-latency (one cycle latency for each operation).

Within the FPU, the 57-bit integer adder and 53-bit integer multiplier are the most computationally intensive blocks and hence we apply the pruning algorithm on these blocks as a basis for our preliminary investigation. In this paper, we use the Kogge–Stone parallel prefix adder^7^ and truncated array multiplier architectures [17] in the FPU.

**Figure 1.**
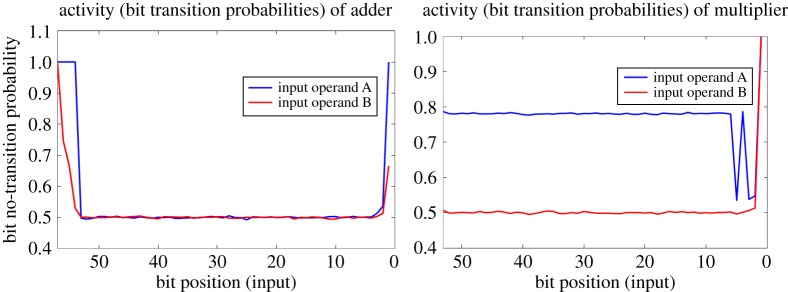
Input traces showing the transition activity in the integer adder and multiplier units of the FPU. The *x*-axis refers to the bit positions of the input bits of the 57-bit adder and 53-bit multiplier. ‘1’ denotes the least significant bit. The *y*-axis corresponds to the probability that a bit-position will have no transition activity (‘0.5’ implies a transition probability of 50%, whereas ‘1’ implies that there is no-transition activity, see text).

Data from simulations of the Lorenz ’96 model were used as input application traces for the annotations needed by the pruning techniques as described in §2*a*. We show the input traces of the integer adder and multiplier units used in our FPU in figure 1. The *x*-axis refers to the bit positions of the input bits of the 57-bit adder and 53-bit multiplier (e.g. the number ‘57’ on the *x*-axis of the integer adder refers to the input most significant bit of the adder, whereas ‘1’ refers to the input least significant bit. The *y*-axis corresponds to the probability that a bit-position has no transition activity when an application trace is run on it (e.g. a value of ‘0.5’ implies a probability of 50% for a transitions between 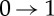 or 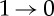, whereas a value of ‘1’ implies that there is no-transition activity in that input bit and it is either a constant ‘0’ or a ‘1’).

As evident from the figure 1, both the adder and the multiplier inputs have a relatively uniform input activity profile across all the input bit positions (one striking difference is the low activity profile of one of the inputs to the multiplier which provides a scope for more aggressive pruning), which puts the onus on the significance values to guide the pruning algorithm. As we use an output significance-driven assignment, the pruning algorithm is likely to converge to bit width reduced blocks as the initial candidate solutions.

In the interests of saving time during pruning and in order to reduce the design space exploration, we start with an initial bit-width-truncated configuration, rather than with pruning from the beginning.

We therefore use the two-step heuristic method identified below.

(i) Apply a coarse-grained *bit width truncation* on the complete circuit graph of the integer adder and multiplier (in this paper, we have used a decreasing step size of 8) and evaluate the application level quality for the obtained designs.
(ii) We then identify the bit-width-truncated circuits, which are closer to the application's error-tolerance threshold, and use them as a starting point to apply the logical pruning algorithm on these reduced circuit graphs to achieve a fine grain exploration and enhance the resource savings further. We term this step as *logic pruning* (LP) which executes the two-step ranking and pruning phases described in §2*a* on the reduced circuit graph annotated with the input traces from the application.

**Figure 2.**
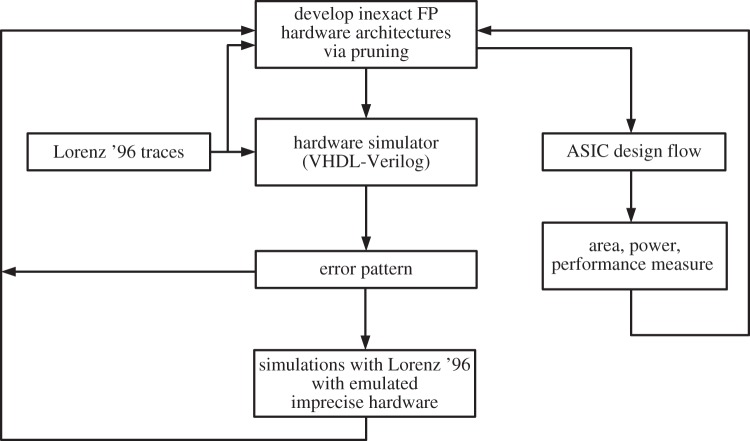
Overview on the framework for designing the optimal inexact FPU. Pruning is performed to develop the inexact hardware architectures using the activity of the Lorenz ’96 traces. Area, power and performance measurements are extracted using the ASIC design flow.^8^ The developed hardware architectures are simulated using the traces, and the inexact floating point co-processor units developed in VHDL-Verilog. Furthermore, traces of simulations with the Lorenz ’96 model are evaluated with the hardware simulator to obtain error pattern for the specific hardware architectures. These error pattern are fed into the emulator that emulates the use of pruned hardware in simulations of the Lorenz ’96 model. The information on possible savings, resulting error pattern and quality of model simulations are used to retune the pruning algorithm.

The derived hardware set-ups are then tested within the Lorenz ’96 model. If the simulations reveal that the errors can be larger, or should be smaller, then the procedure is repeated with adjusted level for the truncation. Several iterations of this process were done for this paper, to reach the optimal hardware set-up. A sketch of the framework and the proposed methodology is presented in figure 2.

## 3. The Lorenz ’96 system

The complexity of^8^ a full weather or climate model together with the need to emulate hardware which is not yet realized as ‘hardware’ prevent us from working with a full weather or climate model. Even restricting ourselves to the dynamical core^9^ of a working model would be a major undertaking. Therefore, we consider a toy model of atmospheric dynamics—the Lorenz ’96 model. The Lorenz ’96 model was proposed in reference [18] and consists of two sets of prognostic variables. The ‘*X*’-variables represent large-scale dynamics of the global atmosphere. These are the quantities we want to predict correctly in global weather and climate simulations. The ‘*Y* ’-variables represent small-scale dynamics of the system that couple to the large-scale variables.

Owing to the coupling of the large- and small-scale variables and the nonlinear behaviour, the Lorenz ’96 system displays multi-scale and chaotic properties that are features of many components of atmospheric dynamics, such as convection, at least to some extent. The system is heavily used to test conceptual ideas for example for data assimilation or parametrization schemes in atmospheric modelling, before complex, global circulation models are investigated [19–22].

### (a) Equations

The large-scale variables form a one-dimensional periodic space. Each large-scale variable couples to a set of small-scale variables that forms a one-dimensional periodic space on its own. We use eight large-scale variables *X*_*k*_ (*X*_*k*−8_=*X*_*k*_=*X*_*k*+8_), and 32 small-scale variables *Y*_*j*,*k*_ (*Y*_*j*−32,*k*_=*Y*_*j*,*k*_=*Y*_*j*+32,*k*_) for each *X*_*k*_.

The Lorenz ’96 system is described by the following set of equations
3.1
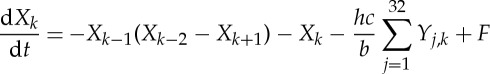
and
3.2
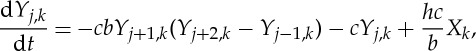
where we use *h*=1, *c*=10, *b*=10 and *F*=20. We use a fourth-order Runge–Kutta scheme to integrate the model in time. For this scheme, the right-hand side of the equations (3.1) and (3.2) needs to be calculated four times per time step, which generates a large part of the computational cost. The size of the time step is 0.0005 model time units. It is generally accepted that one model time unit of the Lorenz ’96 model corresponds approximately to five atmospheric days.^10^ We compare the results of simulations with the full system with results of a reduced system for which the small-scale variables are parametrized (the deterministic scheme in reference [23]):

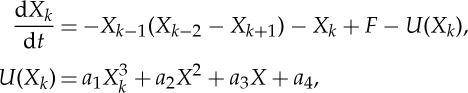
where *U*(*X*_*k*_) tries to mimic the behaviour of the *Y* variables and *a*_1_=−0.00235, *a*_2_=−0.0136, *a*_3_=1.3 and *a*_4_=0.341.

Because the parametrized system has only eight degrees of freedom (compared with the 264 degrees of freedom of the full model), it is much cheaper and can therefore serve as a lower limit for the forecast quality. Simulations with the full system on emulated inexact hardware should always show a higher quality compared with the parametrized system.

### (b) Emulator for inexact hardware

To develop meaningful emulators for the different set-ups for inexact hardware, simulations of the full, unperturbed Lorenz ’96 system are performed and the minimal and maximal values of the input variables for each operation, for which reduced precision is emulated, are measured. Afterwards, the two-dimensional space (one-dimensional if one of the input variables is a constant) between the minimal and maximal values of the two input values of a specific operation is discretized into a grid with 50×50 grid cells. To set up the emulator, we need to assign a specific error value for input variables that fall within a specific grid cell of the grid of input variables. To this end, we calculate at least 20 sets of random input variables that fall within the range of each grid cell and calculate the error each hardware set-up would show for the sets of input variables, using the hardware simulator. Out of the error values calculated for each grid cell, the largest error that is present is stored in a table for which each entry belongs to a specific grid cell. We create such a look-up table for each operation and each hardware set-up. If the emulator is used to mimic the use of a specific inexact hardware for a specific operation within simulations of the Lorenz ’96 model, then the error that is stored in the corresponding look-up table is added to the result of the operation, if the input variables fall into a given grid cell. If the inputs to an operation fall outside of the range of the look-up table the largest error from the entire table is added. To this end, the emulator represents a kind of worst-case scenario for the error induced by the imprecise hardware.

It is known from reference [10] that the calculation of the right-hand side of the small-scale variables is quite forgiving when processing errors are included.^11^ First tests therefore calculate the right-hand side of equation (3.2) on the emulator.

The multiplications that involve constants in equation (3.2) can be done before the time integration is started. When calculating *c*_1_=−*cb*, *c*_2_=−*c* and *c*_3_=*hc*/*b* in advance, we end up with the following seven consecutive operations that are necessary to calculate the right-hand side of equation (3.2), of which three of them are addition or subtraction, and four of them are multiplications:

(i) *r*_1_=*Y*_*j*+2,*k*_−*Y*_*j*−1,*k*_,
(ii) *r*_2_=*Y*_*j*+1,*k*_⋅*r*_1_,
(iii) *r*_3_=*c*_1_⋅*r*_2_,
(iv) *r*_4_=*c*_2_⋅*Y*_*j*,*k*_,
(v) *r*_5_=*r*_3_+*r*_4_,
(vi) *r*_6_=*c*_3_⋅*X*_*k*_ and
(vii) d*Y*_*j*,*k*_/d*t*=*r*_5_+*r*_6_.

Traces of these operations are used to determine the activity values for pruning.

**Figure 3.**
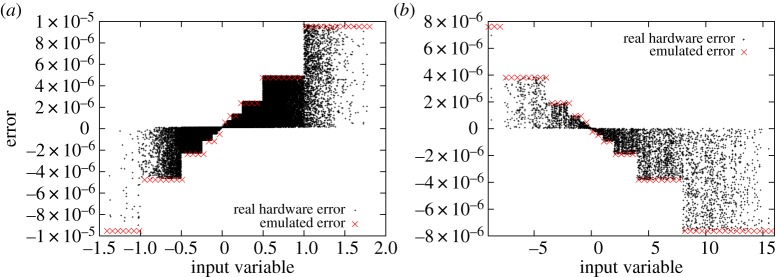
Error pattern of a pruned FP architecture (M4 multiplier block; table 1) and error pattern of the emulator for reduced precision plotted against the variable input parameter. (*a*) Results for multiplication (iv) and (*b*) for multiplication (vi) listed in the text. The input values that were used to generate the error pattern for the real hardware error were taken from a Lorenz ’96 simulation on precise hardware. (Online version in colour.)

Figure 3 shows the error pattern for two operations with one constant input variable for the derived hardware and the emulator. It can be seen that the magnitude of the error is changing stepwise when the exponent of the FP is changing. This is what we would expect when bit width truncation is used.

Eventually, the whole model was put onto the emulator, performing the same steps for each operation as before.

## 4. Results

We present the developed FP architectures in detail and test the quality of the different set-ups when calculating the right-hand side of the equation for small-scale variables in Lorenz ’96 with emulated errors. We discuss the results, characterize the perturbations and decide on four reasonable combinations of adder–subtractor and multiplier blocks for further investigations. These combinations are used to calculate different parts of the code on emulated inexact hardware, to evaluate the different sensitivities to inexact hardware. Finally, we run short- and long-term simulations using different hardware combinations in the different parts of the code as benchmarks, compare the results with precise and parametrized simulations, and discuss possible savings.

### (a) Inexact hardware structures

In table 1, we compare the synthesis results of the pruned FP architectures with the conventional exact FPU using the Nangate 45 nm Open Cell Library (v. 1.3 [24]; slow corner).^12^ We have pruned the adder–subtractor and multiplier integer blocks in different ways. The target is to provide the reader with trends and associated trade-offs, in terms of area, power, performance and impact on the simulation.

**Table 1.** Comparison of the inexact FPU architectures (area, power, speed and error) using the NanGate 45 nm Open Cell Library v. 1.3 [24]. The listed relative maximal and mean errors were calculated over a range of about 350 000 operations. We define a relative error to be the absolute difference between the exact and the imprecise result, divided by the absolute exact result. The listed forecast errors show the mean absolute difference between the large-scale quantities *X*_*n*_ of exact and imprecise simulations of the Lorenz ’96 model after one model time unit, averaged over 5000 simulations. T, Truncation (coarse-grained bit width truncation with eight-bits step size inside the mantissa & each integer adder–subtractor–multiplier). LP, Logic pruning (fine-grained elimination of redundant logic inside each adder–subtractor–multiplier).

					error		
floating point architecture	pruning method	area (umm^2^)	power dynamic (mW)/leakage (uW)	critical path delay (ns)	relative max	relative mean	forecast error after one model time unit
add/subtract	no pruning	6851.63	2.03/110.08	7.16	0	0	0
A1	8-bits-T	5813.96	1.70/92.71	7.02	2.3034×10^−10^	4.2911×10^−14^	6.44×10^−05^
A2	16-bits-T	4939.10	1.44/79.01	6.73	9.4079×10^−08^	1.1350×10^−11^	2.04×10^−03^
A3	24-bits-T	3984.95	1.22/62.72	6.25	1.2355×10^−05^	2.8199×10^−09^	2.03×10^−02^
A4	32-bits-T	3176.30	1.04/49.49	6.01	6.8576×10^−03^	7.2657×10^−07^	8.35×10^−02^
A5	40-bits-T	2157.26	0.71/33.81	5.56	5.7156×10^−01^	1.7536×10^−04^	0.24
A6	41-bits-T	2065.49	0.69/32.22	5.28	6.8555×10^−01^	3.2711×10^−04^	0.27
A7	A6+LP	2026.92	0.73/31.31	5.26	2.2082×10^+00^	2.4724×10^−02^	4.34
A8	A6+LP	2110.44	0.78/32.33	4.89	2.9651×10^+00^	8.5332×10^−02^	4.95
A9	A6+LP	2037.83	0.78/31.46	4.73	8.7366×10^+01^	3.1454×10^−01^	crash
A10	A6+LP	2057.78	0.81/31.96	4.72	1.4998×10^+00^	1.7314×10^−01^	crash
**multiply**	**no pruning**	**14 975.00**	**6.32/209.72**	**5.99**	**0**	**0**	**0**
M1	8-bits-T	11 177.32	4.87/156.87	5.87	1.0523×10^−13^	2.5555×10^−14^	5.55×10^−05^
M2	16-bits-T	8316.22	3.48/118.55	5.48	2.8095×10^−11^	6.5740×10^−12^	1.80×10^−03^
M3	24-bits-T	5483.32	2.32/78.70	5.35	7.0917×10^−09^	1.6835×10^−09^	1.91×10^−02^
M4	32-bits-T	3581.42	1.56/52.64	4.66	1.8000×10^−06^	4.3131×10^−07^	8.13×10^−02^
M5	40-bits-T	1893.12	0.81/27.95	4.07	4.6948×10^−04^	1.1060×10^−04^	0.23
M6	37-bits-T	2563.97	1.10/38.17	4.11	5.7324×10^−05^	1.3815×10^−05^	0.16
M7	M6+LP	1214.55	0.53/17.65	4.83	9.4211×10^−03^	1.4008×10^−03^	0.37
M8	M6+LP	1007.83	0.48/15.65	3.67	2.9802×10^−02^	8.1950×10^−03^	0.59
M9	M6+LP	993.24	0.45/14.40	3.29	5.7110×10^−02^	1.5993×10^−02^	0.80
M10	M6+LP	927.80	0.43/13.97	2.47	1.8180×10^−01^	5.3686×10^−02^	1.25

As expected, with the approach presented in this work, we can achieve between ≈16% and 66% reduction in energy consumption, with corresponding delay and area reductions between ≈2–34% and ≈15–70%, respectively, for the pruned adder–subtractor blocks (i.e. A1–10) w.r.t. the exact implementation. On the other hand, for the pruned FP multiplier blocks (i.e. M1–10), we can achieve energy reductions between ≈23% and 93%, with corresponding delay and area reductions between ≈2–59% and ≈25–94%, respectively. Of course, all savings are at the cost of losing precision when computing FP operations. For instance, in the A6 architecture, the reductions are ≈66%, ≈70% and ≈26% in terms of power, area and performance, respectively, with an associated relative error between 7.6138×10^−10^ and 6.8555×10^−01^. By contrast, in the M10 architecture, the energy and area is reduced by ≈94%, with the corresponding performance improvement of ≈59%, with a relative error bounded from 2.4629×10^−07^ up to 1.8180×10^−01^.

The forecast errors in table 1 refer to the mean, absolute error of the large-scale variables compared with a control simulation on precise hardware. We simulate 5000 short-term forecasts with the Lorenz ’96 model with emulated inexact hardware using the control simulation for initialization. The forecasts are started in intervals of 10 model units of the control simulation. For the FP adder–subtractor or multiplier blocks either the three sums and subtractions or the four multiplications necessary to calculate the right-hand side of the short-term variables (see operations (i)–(vii) in §3*b*) are calculated with emulated errors for the respective inexact hardware.

We evaluate the average forecast error for the large-scale variables after one model time unit (2000 time steps) when comparing to the control run. The forecast errors are increasing with increasing maximal and mean hardware error, as expected. The adder–subtractor blocks with logic pruning produce large forecast errors and even model crashes (for A9 and A10). We attribute this to the inherent set-up of the emulator as it pessimistically adds the largest observed error over an application test bench run to every inexact computation in the emulator to account for the worst-case scenario. This pessimistic approach naturally favours inexact techniques which limit the worst-case errors (e.g. bit width truncation) as opposed to those which lower the average case errors (e.g. logic pruning) as identified in reference [4]. We hope to remedy this, in future work, by injecting observed error distributions in the emulator rather than in worst-case errors. However, the multiplier with logic pruning allows results that are much better.

**Figure 4.**
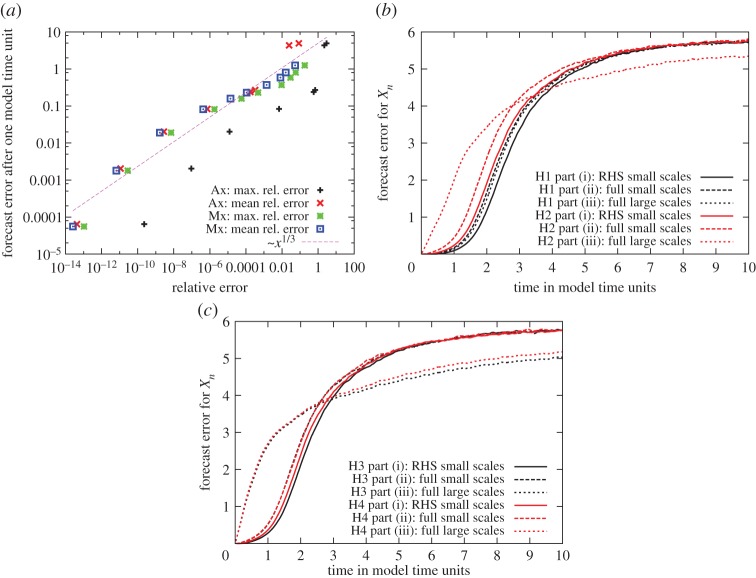
(*a*) Forecast error of the large-scale variables after one model time unit plotted against the maximal or mean relative error for the different FP adder–subtractor and multiplier blocks in table 1. In a rough approximation, the forecast error behaves proportional to *x*^1/3^. (*b*,c) Forecast error plotted against time when using different hardware combinations in different parts of the model. The emulated hardware combinations (H1–H4) are used either to calculate the right-hand side (RHS) or the full equations for the large- and small-scale variables (parts (i)–(iii) mentioned in the text). The forecast error shows the expected behaviour for a chaotic system plotted against time with an exponential growth at the beginning, for which the growth rate is dependent on the magnitude of the perturbation, and a convergence towards a fixed error when the perturbed and unperturbed system become more and more uncorrelated.

Figure 4*a* shows the forecast error plotted against the maximum and the mean relative error of the used hardware. In a rough approximation, the forecast error behaves proportional to the relative error of the simulated hardware to the power of one third (see the ∼*x*^1/3^ function in figure 4*a*).

The different FP adder–subtractor and multiplier blocks in table 1 can be combined arbitrarily, to form a full FPU. We decide on combinations based on the forecast errors after one model time unit that have a similar range. We consider four combinations of FP adder–subtractor and multiplier blocks in the rest of this paper which are listed in table 2. In a first test, we apply these combinations independently to three different parts of the model.

(i) To calculate the right-hand side of the equation for small-scale variables (equation (3.2)).
(ii) To calculate the full dynamics of the small-scale variables (equation (3.2)).
(iii) To calculate the full dynamics of the large-scale variables (equation (3.1)).

**Table 2.** Emulated hardware combinations of adder–subtractor and multiplier blocks used in simulations of the Lorenz ’96 model.

combination of adder–subtractor and multiplier block	adder–subtractor block	multiplier block
H1	A4	M4
H2	A5	M6
H3	A6	M5
H4	A6	M7

Figure 4*b,c* shows the forecast error against time for the different architectures used in the different parts of the model. Given that a model time unit corresponds to approximately five atmospheric days, all simulations in figure 4 appear to have an error that is reasonably small, except for the simulations in which H2, H3 or H4 are used to calculate the large-scale variables. In these simulations, the forecast error is smaller on the long term, because the heavy change in the dynamics of the system leads to a smaller difference for uncorrelated perturbed and unperturbed systems, compared with the difference between two uncorrelated, unperturbed systems. As expected, the error is smaller for part (i) compared with part (ii) when different imprecise architectures are used.

### (b) Benchmark simulations with Lorenz ’96 on inexact hardware and discussion of possible savings

Based on the results of the §3*a*, we perform one simulation for which the H1 architecture is emulated for the whole model integration and three simulations for which the dynamics of the small-scale variables are calculated with the H2, H3 or H4 architecture, whereas the large-scale dynamics are calculated with H1. We use these set-ups to calculate the forecast error in short-term simulations as before and perform additional long-term simulations. We simulate each set-up for 100 000 model time units after spin-up for the long-term simulations.

**Figure 5.**
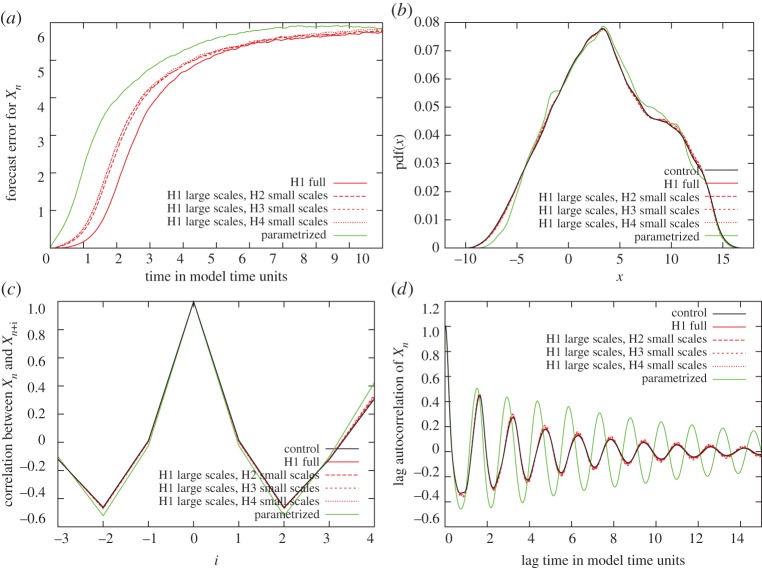
Benchmark simulations with Lorenz ’96: forecast error for the four set-ups of inexact hardware against time for short-term simulations, and PDF, spacial and temporal correlation for the *X*_*n*_ variables in long-term simulations (top left to bottom right). The simulations with emulated inexact hardware give clearly better results than the parametrized simulations, because the forecast errors are smaller in *a*, and the red lines are always much closer to the black lines of the control simulation compared to the green lines in *b–d*.

Figure 5 shows the results for forecast errors against time for the short-term simulations (figure 5*a*), and the probability density function (PDF; figure 5*b*) and spatial and temporal correlation (figure 5*c,d*) of the *X*_*n*_ values for the long-term simulations. The forecast errors for all simulations with inexact hardware are reasonably small, given that a model time unit corresponds to approximately five atmospheric days in terms of predictability and that the quality of a typical weather forecast is declining fast beyond a couple of days of a forecast. For all diagnostics, the simulations with emulated inexact hardware give better results than the simulations in which the small-scale dynamics are parametrized.

We calculate the Hellinger distance, *H*, as a measure of the difference between two PDFs:
4.1
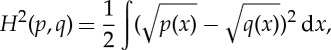
where *p*(*x*) is the PDF of the imprecise or parametrized simulation, whereas *q*(*x*) is the PDF of the control simulation. Table 3 lists the mean of the *X*_*n*_ values and the Hellinger distance for the different set-ups.

**Table 3.** Mean and Hellinger distance of large-scale variables for the long-term simulations with different set-ups. The Hellinger distance quantifies the difference between the PDF of the control simulation and the PDF of other simulations (see equation (4.1)). A large Hellinger distance indicates a large difference of the PDFs.

	mean of *X*_*n*_	Hellinger distance
control	3.77	
H1 full	3.78	1.03×10^−03^
H1 large scales, H2 small scales	3.78	5.36×10^−03^
H1 large scales, H3 small scales	3.80	1.10×10^−02^
H1 large scales, H4 small scales	3.80	8.01×10^−03^
parametrized	3.90	4.49×10^−02^

In summary, the simulations with H1 show that the full model can be calculated with simulated hardware that has 54%, 49% and 16% savings in area, power and delay for the FP adder–subtractor block and 76%, 75% and 22% savings in area, power and delay for the FP multiplier block without serious penalties. When profiling a model simulation of the full Lorenz ’96 model on precise hardware,^13^ it turns out that about 75% of the computational cost, in terms of execution time, is caused by the calculation of the small-scale dynamics, about 19% is caused by the calculation of the large-scale dynamics and about 6% is caused by output and model coordination. The errors stay reasonably small for the set-ups H2, H3 and H4. We therefore conclude that about 75% of the computational cost for the control simulation, in terms of execution time, could be put on hardware that has up to 70%, 66% and 26% savings in area, power and delay for the FP adder–subtractor block and 92%, 92% and 19% savings in area, power and delay for the FP multiplier block.

## 5. Conclusion and future directions

In this paper, we demonstrate the potential utility of inexact hardware for atmospheric modelling. The results show that the Lorenz ’96 model can tolerate the use of inexact hardware in large parts of the model integration without major changes in the forecast quality of weather- and climate-type diagnostics, while benefiting from substantial reductions in the power dissipation and area of the FPU, and improvements to hardware performance. Our results suggest that the motivation behind this paper—to use very efficient but inexact hardware to potentially cope with the ever-increasing power consumption of state-of-the-art supercomputers for modelling weather and climate—is worth investigating and has the potential to lead to a new class of models and hardware for computational fluid dynamics.

The simulations with Lorenz ’96 confirm the result in reference [10] that the different parts of a model for atmospheric dynamics have very different sensitivities to hardware errors. Approaches that take care of the different sensitivities, such as scale separation, are crucially important when calculating a weather or climate model on inexact hardware. Our results suggest that large parts of the Lorenz model can cope with strong errors. However, the Lorenz model is no more than a toy model and can be assumed to be fairly forgiving in terms of inexactness, because it has relaxation terms and a natural scale separation which is not apparent in full atmosphere models. Further tests are needed to verify that an application of inexact hardware to small-scale dynamics of a high-resolution weather or climate model has no negative influence on large-scale dynamics. Further tests are also necessary on the influence of hardware errors on conservation and convergence behaviour and on the sensitivity of different discretization schemes in time and space to hardware errors. The hardware is neither produced nor tested in great detail yet, and the emulator used is still rather crude.

The technique of pruning used in this paper is a relatively easy approach to obtain inexact hardware set-ups with high savings in area, power and performance. While the combination of bit width truncation and logic pruning seems to have already achieved substantial savings (compare M7–10 with M6 in table 1), we anticipate that applying cross-layer inexact techniques through a co-design framework on the lines of reference [11] would further boost the resource savings. As mentioned in §4*a*, the current emulator pessimistically adds the worst-case error observed over a set of test vectors to every computation involving the inexact FPU and hence is inherently biased against the inexact techniques that minimize average error while allowing a small number of fairly large errors. We need to refine the emulator to add the appropriate error distribution as opposed to a single worst-case error value in future work.

We plan to conduct more studies on how to gracefully and efficiently integrate exact and inexact hardware and how to load balance the different parts on parallel machines. Approaches to trade-off exactness against a reduced energy demand should be extended to memory and data storage, because memory bandwidth is a major bottleneck for many atmosphere models. Future work will focus on the use of inexact hardware in larger models, such as the dynamical core of an atmosphere model. To this end, the development of inexact hardware and an appropriate test set-up needs to go hand in hand. A strong cooperation between hardware developers and users is essential.

## Footnotes

1 The performance in high-performance computing is often measured by the number of floating point (FP) operations per second (FLOPS). Today's top supercomputers have performance measured in tens of petaFLOPS—10^16^FLOPS.

2 Inexact computing has since grown as an area of study and innovation, and several papers have been written by a variety of groups including ours; we are citing only those papers that are directly relevant to the techniques used in §2 to induce inexactness in the floating point units, and the reader is referred to the general literature for additional reading on this rapidly evolving area.

3 The authors acknowledge that there is no novelty in the inexact method of choice (probabilistic pruning) in this paper obtained from reference [8] including the scope of its use to the integer unit which constitutes computing the mantissa part of the floating point unit only. However, the novelty is in evaluating the impact of this inexact technique with the overall architecture of a floating point unit in terms of the savings achieved, and its eventual impact on the application level quality in the Lorenz ’96 application (described in §3).

4 A ‘greedy algorithm’ refers to making the locally optimal choices at each step in the hope of finding a global optimum solution. In this context, we are using such a ‘greedy’ approach by substituting the output of a node that is marked for pruning with its most observable value (a 0, 1 or one of the node's inputs) determined through the annotations.

5 This step also includes the healing phase of the pruning algorithm in Lingamneni *et al.*[11].

6 The targeted error bound is determined by the application. In the case that there is no well-quantified error bound, we impose a range of artificial error targets on the pruning algorithm and use the resulting inexact pruned circuits in the application to estimate the error tolerance bounds.

7 A Kogge–Stone adder is a hardware design which is used frequently for sums of binary numbers in state-of-the-art computing [16].

8 The application-specific integrated circuit (ASIC) design flow consists of a methodology and associated sets of tools to synthesize/generate, validate and test hardware architectures. These tools offer the possibility to estimate area, power and performance (circuit delay) before manufacturing the final integrated circuit.

9 The dynamical core refers to the portion of the code that involves fluid dynamical behaviour, without the influence of moisture, clouds, ice and other physical processes and forcings which are dealt with in the ‘parametrization’ portion of the model.

10 The error growth after one model time unit in Lorenz ’96 was estimated to be similar to the error growth in a numerical weather forecast after 5 atmospheric days [18]. However, this number needs to be reduced slightly nowadays, because the forecast quality of weather models has improved (Hannah Arnold 2013, personal communication).

11 A fairly crude emulator that induced random bit flips of one of the bits of the significand into the result of 20% of all FP operations was used to calculate the right-hand side of equation (3.2) without a serious reduction of the quality of the results for large-scale variables in long-term simulations.

12 In this work, we use the slow corner of the Nangate 45 nm Open Cell Library (SlowSlow process, voltage =0.95V and temperature =125^°^C) in order to obtain the area, power and performance measurements.

13 Using the gnu profiler gprof and the Intel Fortran compiler with O3 optimization, and running the code on an Intel i7 CPU.
